# Effect of an app on students’ knowledge about diabetes during the COVID-19 pandemic

**DOI:** 10.1590/1518-8345.5798.3541

**Published:** 2022-05-30

**Authors:** Francisca Diana da Silva Negreiros, Amanda Caboclo Flor, Virna Ribeiro Feitosa Cestari, Raquel Sampaio Florêncio, Thereza Maria Magalhães Moreira

**Affiliations:** 1 Universidade Federal do Ceará, Hospital Universitário Walter Cantídio, Fortaleza, CE, Brasil.; 2 Universidade Estadual do Ceará, Fortaleza, CE, Brasil.; 3 Bolsista da Coordenação de Aperfeiçoamento de Pessoal de Nível Superior (CAPES), Brasil.; 4 Bolsista do Conselho Nacional de Desenvolvimento Científico e Tecnológico (CNPq), Brasil.

**Keywords:** COVID-19, Diabetes Mellitus, Nursing, Students, Nursing, Mobile Applications, Educational Technology, COVID-19, Diabetes Mellitus, Enfermagem, Estudantes de Enfermagem, Aplicativos Móveis, Tecnologia Educacional, COVID-19, Diabetes Mellitus, Enfermería, Estudiantes de Enfermería, Aplicaciones Móviles, Tecnología Educacional

## Abstract

**Objective:**

to analyze the effect of an app on Nursing students’ knowledge about diabetes during the COVID-19 pandemic, as well as their self-assessment and satisfaction level.

**Method:**

a quasi-experimental study carried out with 40 Nursing students from the Brazilian Northeast region. The E-MunDiabetes^®^ app was used to assess the participants’ knowledge at the pre-test, immediate post-test and after 15 days, as well as their self-assessment and satisfaction level in relation to using the app. The analysis was performed by means of descriptive and inferential statistics (binomial test, Intraclass Correlation Coefficient and Wilcoxon’s test).

**Results:**

the comparison of the medians of correct answers in the three periods revealed a significant increase in the post-test. The self-assessment and satisfaction items presented an Agreement Index > 80%, with a total Agreement Index of 96.3% and an Intraclass Correlation Coefficient of 0.91.

**Conclusion:**

the app was considered satisfactory and promoted a significant increase in the students’ knowledge, therefore being suitable for its intended use.

## Resumo

**Objetivo:**

analisar o efeito de um aplicativo no conhecimento de estudantes de Enfermagem sobre diabetes durante a pandemia da COVID-19 bem como sua autoavaliação e satisfação.

**Método:**

estudo quase experimental realizado com 40 estudantes de Enfermagem da região nordeste, Brasil. Foi utilizado o aplicativo E-MunDiabetes^®^ para avaliar o conhecimento dos participantes pré e pós-teste imediato e após 15 dias, bem como a autoavaliação e a satisfação relacionadas ao uso do aplicativo. A análise foi realizada por meio de estatística descritiva e inferencial (teste binomial, coeficiente de correlação intraclasse e teste de Wilcoxon).

**Resultados:**

a comparação das medianas de acertos nos três períodos relevou aumento significativo no pós-teste. Os itens da autoavaliação e satisfação apresentaram índice de concordância >80%, com índice de concordância total de 96,3% e coeficiente de correlação intraclasse de 0,91.

**Conclusão:**

o aplicativo foi considerado satisfatório e promoveu aumento significativo do conhecimento dos estudantes, estando, portanto, adequado para o uso.

## Resumen

**Objetivo:**

analizar el efecto de una aplicación sobre el conocimiento de estudiantes de Enfermería sobre diabetes durante la pandemia de COVID-19 y su autoevaluación y satisfacción.

**Método:**

estudio cuasiexperimental realizado con 40 estudiantes de Enfermería de la región nordeste de Brasil. Se utilizó la aplicación E-MunDiabetes^®^ para evaluar los conocimientos de los participantes pretest y postest inmediato y después de 15 días, así como la autoevaluación y la satisfacción relacionada con el uso de la aplicación. El análisis se realizó mediante estadística descriptiva e inferencial (prueba binomial, coeficiente de correlación intraclase y prueba de Wilcoxon).

**Resultados:**

la comparación de las medianas de aciertos en los tres períodos reveló un aumento significativo en el postest. Los ítems de autoevaluación y satisfacción mostraron una tasa de acuerdo >80%, una tasa de acuerdo total del 96,3% y un coeficiente de correlación intraclase de 0,91.

**Conclusión:**

la aplicación fue considerada satisfactoria y promovió un aumento significativo en el conocimiento de los estudiantes, por lo tanto, es apta para ser utilizada.

**Highlights**

(1) First app aimed at teaching students about diabetes care.

(2) The pre- and post-test comparisons indicate that the app is effective in terms of acquisition and retention.

(3) The self-assessment and satisfaction indices presented high values and agreement.

## Introduction

Since the first global records on COVID-19, a high percentage of those affected had Diabetes Mellitus (DM), especially in the cases with a fatal outcome. Globally, COVID-19 became a major concern for the DM community^1^. At the same time, it triggered concerns and efforts in knowledge construction for governmental entities, teaching and research institutions, academic classes and health professionals, with a view to its management and coping. It so happens that, in line with the advances in knowledge about COVID-19, health professionals have faced difficulties in clinical management and in supporting the heavy workload in the health systems^2^. 

In an attempt to alleviate the burden of front-line health professionals in coping with the pandemic, managers sought help from health students, particularly in the Nursing area^3^ ^-^ ^4^. The global crisis installed by COVID-19 requires Nursing professionals and students to improve their knowledge about the disease and adapt to new and rapid teaching-learning methods, which have exerted a positive impact on knowledge construction and dissemination to better target the professional practice towards the urgent health needs imposed by the emerging pandemic situation^5^.

Understanding as a premise that Nursing students’ learning requires innovation, subsidies were sought in the Problem Based Learning (PBL) method to develop the proposal described in this study. Thus, the current study adds knowledge to the literature, as effective use of PBL embedded in mobile devices provides consistent information to enrich the learning experience, promoting strong practical motivation and cognitive stimulation for creative solutions to problem cases^6^. It is believed that PBL represents the teaching-learning perspective anchored in knowledge (re)construction, where the process is centered on the students and on their ways of interpreting, researching and seeking answers to solve a case or answer questions. Furthermore, it may present favorable results in the remote education period, due to the suspension of school activities during the pandemic^7^. 

The COVID-19 pandemic is a public health event and, even in the face of the administration of anti-COVID-19 vaccines, existing and available at the moment, it is also necessary to implement preventive measures and education in health^8^. In view of this, it is indispensable that Nursing students are trained and updated regarding education and reinforcement of guidelines on COVID-19 management together with DM control, in line with the scientific updates released constantly and on a large scale through digital technologies.

In this scenario, the use of educational technologies in the digital environment stands out for the possibility of promoting communication and meeting the receiver’s needs more satisfactorily, providing reliable information for health maintenance, thus fostering technological advance in favor of the care process^9^. Therefore, it is understood that digital technologies emerge to mediate the teaching-learning process and to make teaching flexible in order to overcome geographical barriers and allow for dynamic learning^10^. Apps stand out among the educational digital technologies.

A number of studies on the use of apps for Nursing students’ teaching and learning, with approaches in different care contexts, have shown positive effects on knowledge acquisition^11^ ^-^ ^13^. A randomized clinical study showed that apps can facilitate the contemporary teaching-learning process, calling upon professionals to develop, validate and use teaching tools^14^. 

In this perspective, it is up to nurses to develop research studies based on a broad theoretical Nursing framework and that include the production of apps for different care dimensions in the current conditions of the health systems^9^. It is noteworthy that there is currently no app about diabetes education during the pandemic imposed by the new coronavirus. In view of this, the “E-MunDiabetes^®^” app on the care of people with DM in global emergencies, such as the COVID-19 pandemic, was developed and validated by judges in the areas of Nursing and Informatics. The body of professionals who participated in face and content validation and in the technology usability assessment considered that the app is adequate^15^. 

In relation to apps with an emphasis on the theme of diabetes, there were no studies on this type of tools specifically designed to improve the knowledge of Nursing students or of those attending courses in the health area. This research aims at filling this gap and at developing, in Nursing students, the understanding of the concepts linked to the new coronavirus, as well as informing about diabetes care during the pandemic. Thus, the objective of the current study is to analyze the effect of an app on Nursing students’ knowledge about diabetes during the COVID-19 pandemic, as well as their self-assessment and satisfaction level.

## Method

### Type of study

A quasi-experimental and non-randomized study of the before-and-after type. 

### Study locus, period and population

The study was carried out between August and September 2021 in a public Higher Education Institution in the northeastern Brazil. The study population consisted of students from the eighth (n=27) and ninth (n=30) semesters of the undergraduate Nursing course at the aforementioned university, totaling 57 students. 

### Sample definition

The study sample was calculated from the formula for studies with comparative groups, as follows: n = (p1.q1+p2.q2).(Zα/2+Zβ)^2^ / (p1-p2)^2^, where: n=sample size; p1=estimated percentage of students who took the pre-test and may have increased their knowledge (p1=0.50); q1=p1 complement (q1=0.50); p2=estimated percentage of students who took the post-test and may have increased their knowledge (p2=0.80); q2=p2 complement (q2=0.20); Zα/2=significance level established (1.96); and Zβ=test power established (0.84).

Thus, the sample was initially calculated with 36 students. The students included were those with an email address, telephone number and availability to participate in the study during extracurricular hours. The students that did not answer the invitation within 10 days were excluded. The participants were selected for convenience and based on the listed criteria. Among the 57 students, 15 did not answer the invitation and two did not complete the required stages (pre- and post-test). Thus, 40 Nursing students comprised the study sample. 

### Data collection instruments

Three collection instruments were used to obtain the data, namely: 1) A questionnaire containing the sociodemographic variables (identification of the Nursing students, experience in care and in the use of the Internet and of mobile apps as learning tools); 2) The Quiz instrument to perform the knowledge assessment test; and 3) A questionnaire for self-assessment and satisfaction regarding the app.

The Quiz instrument was inserted into the app to assess the level of prior knowledge and that acquired immediately after using the technology, being applied in the pre-test (P0), immediate post-test (P1) and late post-test after 15 days (P2). This instrument addressed questions about prevention and care measures during the COVID-19 pandemic in people with DM, and its content was based on the diverse evidence from the Guidelines of the Brazilian Society of Diabetes^16^ ^-^ ^17^, the World Health Organization^18^ and the Association of Diabetes Care & Education Specialists^19^. 

The Quiz contains a clinical case and ten multiple-choice questions with four answer options (a, b, c and d), of which only one is correct. The question items are programmed to change positions with each user access, so that the answers are not memorized. The clinical case contained a problem situation, with diverse information related to a female patient with a flu-like syndrome and comorbidities, treated in primary care. The clinical case and the ten questions were based on the diverse information contained in the virtual educational technology developed and were validated by a group of nurses who are experts in diabetes care and in the development of educational technologies (n=29). The number of evaluators followed Pasquali’s framework^20^, which states that a number of six to twenty specialists is recommended for the validation process.

The questionnaire regarding self-assessment and satisfaction level related to the use of the “E-MunDiabetes^®^” app contains twenty items, subdivided into four domains: organization, writing style, appearance and motivation. The following scale was adopted to assess the items: 0: I disagree; 1: I partially agree; and 2: I totally agree. The instrument’s total score is calculated as the sum of all the domains. In addition to that, the following questions were asked: How often have you used this app? How much time did you spend using this app? Would you like to suggest something else to improve the app? Did you have any difficulty using it? Would you like to suggest something to improve it? Did you have any difficulties using it, if so, which one? 

### Data collection

Data collection was carried out in two phases, both virtually, in response to the isolation condition imposed by the COVID-19 pandemic. The first phase consisted in recruiting the students through an electronic invitation, informing them about conduction of the research. The following documents were initially sent to the students who agreed to participate: invitation letter with the information and the link to download the “E-MunDiabetes^®^” app with the Quiz instrument for the pre-test and the immediate post-test, as well as the REDCap link to access the Free and Informed Consent Form (FICF) and the data collection instruments for characterization and for self-assessment and satisfaction with the app. 

The first phase of the educational intervention consisted of four stages, described below:

1. Downloading the “E-MunDiabetes^®^” app and signing the FICF. This stage consisted in recruiting the students through an electronic invitation with diverse information about the research. The following documents were sent to the students who agreed to participate: invitation letter with the information and the link to download the “E-MunDiabetes^®^” app, the Quiz instrument for the the pre-test and the immediate post-test, and the REDCap link to access the FICF and the data collection instruments (characterization, self-assessment and satisfaction with the app).
  “E-MunDiabetes^®^” is a tool that was elaborated and validated based on the guidelines for diabetes management and on scientific references that aims at assisting students in the development of education on diabetes during world emergencies, such as COVID-19. The app is compatible with smartphones and tablets that operate with the iOS and Android technologies and can be found from the search tools of these platforms, by typing the name “E-MunDiabetes^®^”^15^. For its download, the user needs Internet access, although the app will be available for offline use after saving it to the smartphone or tablet memory.
  The “E-MunDiabetes^®^” app consists of five screens. The initial one welcomes the user and provides diverse information about the app’s structure, content and navigation. The second screen is called “*Informações gerais sobre a COVID-19*” (“General information about COVID-19”). The third screen is called “*Educação diabetológica na era COVID-19*” (“Education on diabetes in the COVID-19 era”) and corresponds to the practice of the seven self-care behaviors in diabetes defined by the Association of Diabetes Care & Education Specialists^19^, with emphasis on the prevention and care measures against the coronavirus. From the fourth screen, the “*Estratificação de caso potencialmente suspeito de síndrome gripal/COVID-19*” (“Stratification of a potentially suspected flu-like syndrome/COVID-19 case”) functionality is displayed. The fifth screen has the “*QUIZ: Teste seus conhecimentos sobre prevenção e cuidado da COVID-19 em pessoas com diabetes*” (“QUIZ: Test your knowledge about COVID-19 prevention and care in people with diabetes”) functionality, which corresponds to the instrument to test the improvement in the students’ knowledge.
2. Answering the characterization questionnaire and pre-test quiz. When they installed the app, the students were directed to the Quiz instrument and it was only after completion that they were granted access to its content. Immediately after handling the technology, the students answered the Quiz. At the end, they were asked to enter their name and email address. From then on, both the students and the researcher were emailed the number of correct and incorrect answers per question. However, the template was only released after completing the immediate Quiz embedded in the app. Upon receiving the answers, the researcher filled in the result of each question on the REDCap platform, enabling continuity of each student’s participation on the platform.
3. Accessing the theoretical contents to deepen knowledge about COVID-19 and diabetes care in pandemic times: texts, PDF articles, links to websites, videos, the functionality on risk stratification and courses of action to be taken, and completion of the immediate post-test Quiz were made available.
4. Answering of the questionnaire for self-assessment and satisfaction with the technology: the students’ suggestions were analyzed and accepted when pertinent.
  The second data collection phase consisted of applying the late post-test after 15 days (P2), on the sixteenth day since answering the immediate post-test. The invitation letter and the late post-test elaborated in *Google Forms* were sent (the same instrument of the pre-test and immediate post-test was applied at P2). The literature considers that there is a decline in knowledge and skills over time^21^. Thus, verification occurred on the 16^th^day, considering that results obtained immediately after educational strategies can be influenced by short-term memorization, which requires a second verification after some time interval to investigate apprehension of the content over the days.
  A maximum period of ten days was determined to return the evaluation, being extended for the same period. Five days after sending the material, the researcher forwarded a reminder by email and telephone, in order to reinforce completion of the instruments and confirm scheduling of the late post-test.

### Study variables and data treatment and analysis

The variables were stored in an electronic database created in the Microsoft^®^Excel program, using the double check and typing technique to reduce possible errors in transcription of the information. Subsequently, the database was transferred to the Statistical Package for the Social Sciences, version 23.0, for further analysis, and presented in graphs and tables. After verifying data normality with the Kolmogorov-Smirnov test for independent samples, the qualitative variables were expressed as absolute and relative frequencies; for the quantitative variables, the medians were calculated.

For self-assessment and satisfaction with the app, a minimum Agreement Index (AI) of 0.80 was adopted^22^. The binomial test, with a significance level of p>0.05, was also employed to verify agreement proportion equal to or greater than 80%, and the Intraclass Correlation Coefficient (ICC) was used as a reliability measure, considering values < 0.40 as low reliability, from 0.40 to 0.75 as moderate reliability, and values > 0.75 as excellent reliability^23^. 

The outcome adopted was learning (acquired knowledge) after handling the app from the comparison of the tests performed. The literature reveals that knowledge means remembering specific facts or the ability to apply specific facts to solve problems or issue concepts with the understanding acquired about a given event^24^.

Knowledge about COVID-19 and diabetes was analyzed in time frames: pre-test (P0 - Immediately before using the app), immediate post-test (P1 - Immediately after using the app) and late post-test (P2 - 15 days after using the app). The Wilcoxon test was performed to compare the variables with asymmetric distribution. The comparison of the medians at the three moments was performed by means of the Friedman test, and the McNemar test was employed to compare the proportions of correct answers per question. A 95% confidence interval was considered, as well as statistical significance when p<0.05. 

### Ethical aspects

This study was approved by the Research Ethics Committee of the State University of Ceará, under opinion No. 4,671,477. All the recommendations set forth in Resolution 466/2012 of the Ministry of Health referring to research involving human beings were respected.

## Results

A total of 40 Nursing students participated in the study. Most of them were female (33; 82.5%), with a minimum age of 21 years old, a maximum of 45 and a median of 23; they had no partners (32; 80.0%) or children (37; 92.5%) and no paid activity (38; 95.0%), reporting not having own incomes (32; 80.0%). Family income was less than 2 minimum wages (21; 52.5%), with a salary corresponding to R$ 1,100.00. Regarding the academic data, most of them were attending the ninth semester of the undergraduate course (25; 62.5%), 24 (60%) were scholarship holders, 32 (80%) participated in a research group and 25 (62.5%) had experience in the care of people with diabetes. The students stated not having attended any course on diabetes (33; 82.5%) or on COVID-19 (27; 67.5%).

As for Internet access, they have broadband (38; 95%) and mobile (39; 97.5%) services at their homes. They have a computer (39; 97.5%) and a cell phone (40; 100.0%) at their homes. They had diverse experience in using digital teaching technologies (35; 87.5%). In relation to cell phones, they use them to learn content of the undergraduate course (33; 82.5%) and they believe that they can be useful to learn new knowledge (40; 100.0%). Regarding use of the “E-MunDiabetes^®^” app, the participants accessed the technology with a frequency of 1 to 3 times a day (31; 77.5%) and for 1 to 2 hours a day (38;95%).

Table 1 shows the comparison of the correct answers and the percentage differences, by question, in the pre-test and in post-tests 1 and 2, with median increases of 6.0, 7.0 and 8.0, respectively. There was an increase in the number of correct answers for all questions when the percentage difference between P1 and P0 and between P2 and P0 was calculated, being significant for questions two (p<0.001), three (p<0.001), four (p=0.031), six (p<0.001), seven (p<0.001), eight (p=0.039) and nine (p=0.017).

**Table 1:** Correct answers and percentage differences for each question in the pre-test and in post-tests 1 and 2. Fortaleza, CE, Brazil, 2021, N=40

Questions	Pre-test f (%)	Post-test 1 f (%)	Post-test 2 f (%)	PD1^*^	PD2^†^	PD3^‡^	p^§^
Q1	29 (72.5)	35 (87.5)	35 (87.5)	+15.0	0	+15.0	0.180
Q2	11 (27.5)	19 (47.5)	26 (65.0)	+20.0	+17.5	+37.5	0.001
Q3	09 (22.5)	14 (35.0)	28 (70.0)	+12.5	+35.0	+47.5	0.001
Q4	33 (82.5)	39 (97.5)	39 (97.5)	+15.0	0	+15.0	0.031
Q5	21 (52.5)	32 (80.0)	25 (62.5)	+27.5	-17.5	+10.0	0.424
Q6	07 (17.5)	19 (47.5)	22 (55.0)	+30.0	+7.5	+37.5	0.001
Q7	36 (90.0)	40 (100.0)	40 (100.0)	+10.0	0	+10.0	0.001
Q8	32 (80.0)	40 (100.0)	39 (97.5)	+20.0	-2.5	+17.5	0.039
Q9	21 (52.5)	31 (77.5)	33 (82.5)	+23.0	+5.0	+27.0	0.017
Q10	28 (70.0)	30 (75.0)	32 (80.0)	+5.0	+5.0	+10.0	0.388
Minimum	0	05	05	-	-	-	-
Maximum	08	10	10	-	-	-	-
Median	6.0	7.0	8.0	-	-	-	-
p (25-75)	5.0-7.0	7.0-8.0	7.0-9.0	-	-	-	-
p-value^||^	0.037	0.001	0.001	-	-	-	-

^*^Percentage Difference between P1 and P0; ^†^Percentage Difference between P2 and P1; ^‡^Percentage Difference between P2 and P0; ^§^McNemar test, using binomial distribution. The percentage differences with gray marks represented an increase in the number of questions; ^||^Kolmogorov-Smirnov test; p (25-75): 25-75 percentile

**Figure 1:**
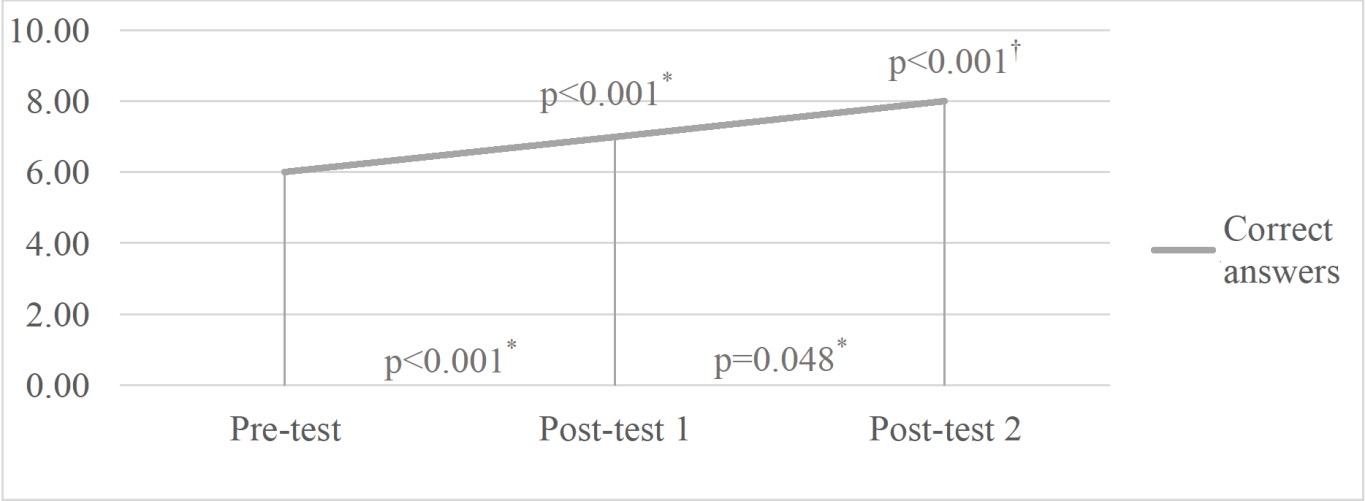
Comparison of the medians of correct answers at the three moments (P0, P1 and P2). Fortaleza, CE, Brazil, 2021 N=40

^*^P0-P1, P1-P2 and P0-P2 = Wilcoxon’s Test; ^†^P0-P2 = Friedman’s Test

Figure 1 shows the comparison of the medians of correct answers in the pre-test and in post-tests 1 and 2, with significant increases between the pre-test and post-test 1 (p<0.001), post-tests 1 and 2 (p=0.048) and pre-test and post-test 2 (p<0.001), shown by Wilcoxon’s test, as well as across the three periods (p<0.001), by means of Friedman’s test.

The participants’ self-assessment and satisfaction with the app showed an AI value > 80%, with an overall AI of 96.3%, meaning significant agreement among the students. The overall ICC was 0.91 (Table 2). The suggestions made by the students were analyzed and accepted, when pertinent.

**Table 2:** Self-assessment and satisfaction regarding the “E-MunDiabetes^®^” app. Fortaleza, CE, Brazil, 2021 N=40

Items		I disagree f (%)	I partially agree f (%)	I totally agree f (%)	AI^*^ (%)	p^†^	ICC^‡^
**First domain - Organization**					95.8		
1.1	Did the app opening draw your attention?	3 (7.5)	10 (25.0)	27 (67.5)	92.5	0.323^§^	0.73
1.2	Is the content sequence adequate?	1 (2.5)	1 (2.5)	38 (95.0)	97.5	0.399	
1.3	Is the app structure organized?	1 (2.5)	5 (12.5)	34 (85.0)	97.5	0.399	
**Second domain - Writing style**					95.8		
2.1	Are the phrases easy to understand?	2 (5.0)	12 (30.0)	26 (65.0)	95.0	0.601^§^	0.74
2.2	Is the written content clear?	1 (2.5)	8 (20.0)	31 (77.5)	97.5	0.399	
2.3	Is the text interesting?	2 (5.0)	9 (22.5)	29 (72.5)	95.0	0.601^§^	
**Third domain - Appearance**					97.5		
3.1	Are the illustrations clear?	1 (2.5)	1 (2.5)	38 (95.0)	97.5	0.399	
3.2	Do the illustrations complement the text?	1 (2.5)	2 (5.0)	37 (92.5)	97.5	0.399	0.85
3.3	Are the screens or sections organized?	1 (2.5)	8 (20.0)	31 (77.5)	97.5	0.399	
**Fourth domain - Motivation**					96.1		
4.1	Will anyone who reads the content of this app understand what it is about?	5 (12.5)	15 (37.5)	20 (50.0)	87.5	0.000^§^	0.79
4.2	Are you motivated to read the app’s content to the end?	2 (5.0)	14 (35.0)	24 (60.0)	95.0	0.601^§^	
4.3	Does the educational material address issues necessary for people with diabetes during the COVID-19 pandemic?	-	6 (15.0)	34 (85.0)	100.0	0.129	
4.4	Did the app improve your confidence in caring for people with diabetes during the COVID-19 pandemic?	1 (2.5)	15 (37.5)	24 (60.0)	97.5	0.399	
4.5	Was the quiz suitable for the app?	-	8 (20.0)	32 (80.0)	100.0	0.129	
4.6	Are the ten questions (quiz) of the app easy?	-	24 (60.0)	16 (40.0)	100.0	0.129	
4.7	Are the complementary readings indicated in the app appropriate?	2 (5.0)	5 (12.5)	33 (82.5)	95.0	0.601^§^	
4.8	Has your knowledge increased by using the app?	1 (2.5)	7 (17.5)	32 (80.0)	97.5	0.399	
4.9	Could you have used the app better?	3 (7.5)	21 (52.5)	16 (40.0)	92.5	0.000^§^	
4.10	Did the app meet your expectations?	3 (7.5)	6 (15.0)	31 (77.5)	92.5	0.323^§^	
4.11	Is the app’s theme relevant?	-	-	40 (100.0)	100.0	0.129	
**Total**					96.3	-	0.91

^*^AI = Agreement Index; ^†^p = Binomial test; ^‡^ICC = Intraclass Correlation Coefficient; ^§^The alternative hypothesis states that the proportion of cases in the first group is < 0.8

The Nursing students appraised the app positively, considering that it has clear information, which meets the target audience’s needs and favors learning, being suitable for circulation in the scientific environment.

## Discussion

The current study showed that the use of the “E-MunDiabetes^®^” app by Nursing students resulted in a significant increase in knowledge about diabetes during the COVID-19 pandemic and that there was high agreement of satisfactory answers to the items related to self-assessment and satisfaction with the use of this technology.

The opening of communication channels in health through information culminates in a new learning proposal, which is more active and participative in a technological era that evidences interactivity^25^. In this context, the use of “E-MunDiabetes^®^”, developed from the active Problem Based Learning method, arises from the need to build bridges between the theoretical and scientific knowledge about diabetes care in the pandemic context imposed by the new coronavirus. This method is employed by several authors who seek holistic and dynamic learning, and is indicated as an effective and highly efficient teaching approach widely applied in educational systems in several countries^26^.

Thus, using validated techniques such as Problem Based Learning, which works in knowledge construction in the face of the discussion of a problem, the scholars argue that the remote learning processes in force, with the social isolation measures proposed, fosters the use of varied active methodologies, due to the repercussions caused by the new coronavirus pandemic. In this context, widely used in the practical field as a method to approach a clinical condition in a unique way, the PBL teaching-learning strategy adapts to the conditions recommended during the pandemic and contributes an adapted resource to promote self-knowledge and the acquisition of interactive knowledge in favor of qualifying the care process^27^.

The study in question revealed a significant increase in the number of correct answers between the pre-test, the immediate post-test and the late post-test after 15 days. Similarly, a quasi-experimental research study of the pre- and post-test type with a single group showed a significant increase in the knowledge variable of 39 Nursing students (p<0.001) when using the Nursing Education Progressive Web Application (NEPWA)^12^. 

Similarly, a quasi-experimental study carried out in southern Brazil detected that there was a substantial statistical difference (p<0.001) in the pre-test and in the post-test correlations, indicating a significant knowledge gain in the Nursing students after using the app^6^. Accordingly, a randomized experimental research study with pre- and post-test conducted in Taiwan with 100 Nursing students used a mobile app for clinical care learning and detected that the experimental group had significantly higher knowledge scores and greater satisfaction levels than the control group^13^. There were correlated findings in a controlled experimental study conducted in Turkey with 122 Nursing students, which pointed out that the post-test of the experimental group that used an app on injection practices had a positive effect on the knowledge levels (p<0.05)^11^. 

The current study revealed an increase in the medians of correct answers from P0 to P2. These same results were found in a randomized controlled clinical trial conducted in northeastern Brazil, which pointed out that the mean of the scores after the intervention increased, especially in the intervention group, showing an effect on the knowledge of students who used an app on therapeutic communication^14^. 

Nurse educators and researchers should collaborate in the development of virtual learning resources to support Clinical Nursing teaching^13^. Given the suggestions, it is recommended, in a future time, to verify the repercussions of using the app in the clinical practice on various health issues and with larger samples and longer follow-ups^14^.

Not all studies with apps to mediate the teaching-learning process had similar methodological designs (some were randomized and others were not), or similar sample sizes; however, they showed a significant improvement in the students’ performance when handling educational apps^6^ ^,^ ^11^ ^,^ ^13^ ^-^ ^14^ ^,^ ^28^. Thus, the literature corroborates the positive influence of this type of technology on Nursing students’ knowledge, when compared to the traditional teaching method. Therefore, it is necessary to design more effective learning materials in digital environments that may encourage learning.

Therefore, it is reiterated that the use of mobile apps plays an essential role as a learning tool for nurses and Nursing undergraduates, enhancing the clinical practice^6^. It can be inferred that the “E-MunDiabetes^®^” app had an effect on Nursing students’ learning, which can have repercussions on the clinical practice for people with diabetes in times of worldwide emergencies, such as the COVID-19 pandemic. 

In this study, the values of the the self-assessment and satisfaction with the app items were adequate, ensuring quality of the technology. Among the items evaluated, it was highlighted that the structures and screens are organized and that the content is clear, interesting and relevant, in addition to having provided more confidence and increased knowledge about the theme. Similarly, a research study conducted in Indonesia showed that the Nursing students were satisfied when using the app in their learning process^12^. In another study, the Nursing students stated that use of the technology increased their motivation and self-confidence and reduced their concerns^11^. 

Similarly to our findings, in a research study conducted in the Philippines on the evaluation of an educational app, the Nursing students attested to its quality through parameters such as ease of use, organization of the content and structuring of the screens. Therefore, based on the results evidenced, the students considered the app developed as an acceptable, reliable and effective tool to be used by nurse educators and Nursing students, in order to improve and enhance the quality of Nursing education^28^. 

In this premise of enhancing undergraduate learning, diabetes educators need to appropriate such technologies, assisting in the dissemination of appropriate and reliable information in learning. In studies on the theme, it can be seen that the use of mobile apps plays an essential role as a learning tool for nurses and Nursing undergraduate students, in addition to facilitating the care provided to the patients in self-management of their disease by disclosing the diverse information acquired within the scope of the technology used^6^ ^,^ ^29^.

In this understanding, the app is configured as a viable alternative for active in-person and/or remote teaching and learning, in an instrument for training students, favoring the discovery of a new knowledge source^30^. Among these areas in which education can be highlighted, the theme of COVID-19 prevention and care in people with diabetes can enable improvements in the Nursing students’ knowledge, as management of people with this disease needs to be grounded on scientific knowledge, under penalty of causing deterioration or irreversible complications in the individuals.

It is worth noting that the production of updated information technologies on topics of global importance enhances a safer role of the students in the fight against the pandemic, with the use of diverse knowledge acquired through digital educational technologies in health promotion and education actions^31^. Accepting the diverse evidence set out in this “E-MunDiabetes^®^” mobile app, Nursing students can more quickly and easily find diverse information about the new coronavirus, the main self-care behaviors in diabetes, severity stratification of the respiratory syndrome and its proper management according to the severity of the case, in addition to a quiz with ten questions, followed by the information suggested for the course of action, aiming at knowledge acquisition by Nursing professionals.

In the health education field, the use of mobile apps is relevant in the teaching-learning process in Nursing undergraduate studies, as it provides opportunities for the exchange of experiences and information between individuals with different realities, expanding access to the content, enabling engagement, limiting geographical barriers and adapting to specific realities^32^. With regard to the Nursing area, the “E-MunDiabetes^®^” app constitutes technological innovation in health, as it turns out to be an app grounded on the needs of compiling diverse information based on scientific recommendations. In addition, this app has added basic and advanced operational functions to enable the user to employ a contemporary, valid and satisfactory product. 

The following stand out among the study limitations: the fact that the students were only followed-up for 15 days, due to the need to finish the research, and the difficulty presented by some students to download the link to access the app, which resulted in the withdrawal of some participants.

As for the implications for the advancement of scientific knowledge, it is believed that “E-MunDiabetes^®^” is a technological innovation for health because it is the first mobile app produced in Brazil that is based on a methodological and quasi-experimental study, aimed at Nursing students and enabling quick access to accurate information about the new coronavirus consonant with the care of people with diabetes. Therefore, it is an updated scientific, educational and professional tool that assists both students and nurses, anywhere and 24 hours a day, favoring the apprehension of diverse scientific knowledge and clinical reasoning. In addition, this app may come to contribute to the routine of Nursing teaching and practice since, considering the complexity of the contents, it offers Nursing students and professionals easy access to an updated tool that will guide them in performing diabetes care.

## Conclusion

This study allowed evidencing a statistically significant increase in the knowledge of Nursing students who used the “E-MunDiabetes^®^” app related to diabetes care during the COVID-19 pandemic. The results of the study support the finding that, at the end of the intervention, there was an improvement in learning through the increase in the median of correct answers in the pre-test, immediate post-test and late post-test. 

In addition, the self-assessment and satisfaction with the app items presented significant agreement among the participants. Elaboration and subsequent use of this technology may come to fill a global knowledge and market gap. It was verified that the students’ knowledge remained higher even after fifteen days after using the app, confirming the effectiveness of this teaching instrument. 

In a future time, it is recommended to verify the outcomes of using the app in the practice and in different performance settings, together with a higher number of participants and with an extended longitudinal follow-up 
